# Closing the policy gap in diabetes care for individuals with advanced CKD

**DOI:** 10.1111/dme.15381

**Published:** 2024-06-10

**Authors:** Hellena Hailu Habte‐Asres, Miranda Rosenthal, Dorothea Nitsch, David C. Wheeler

**Affiliations:** ^1^ Florence Nightingale Faculty of Nursing, Midwifery and Palliative Care King's College London London UK; ^2^ Royal Free London, NHS Foundation Trust London UK; ^3^ King's College Hospital London UK; ^4^ Department of Non‐Communicable Disease Epidemiology, Faculty of Epidemiology and Population Health London School of Hygiene & Tropical Medicine London UK; ^5^ UCL Department of Renal Medicine University College London London UK

**Keywords:** diabetes model of care, integrated diabetes CKD care, joint diabetes CKD clinic

## Abstract

**Aim:**

The co‐existence of diabetes and CKD poses significant challenges to healthcare systems, current frameworks often inadequately address the complex needs of individuals with both conditions. Recognising these gaps, we introduced a new diabetes care model for people with advanced CKD in renal satellite units.This paper aims to evaluate this new diabetes model care.

**Method:**

We conducted a prospective audit of a new integrated diabetes kidney care model. Data were presented as mean ± SD or counts/percentages, and pre‐ and post‐intervention differences were assessed using paired samples t‐tests.

**Results:**

A total of 291 individuals with diabetes and advanced CKD stages 4 or 5, or undergoing haemodialysis, were included. The mean age was 68.5 (±13.0) years, 58.4% were males. Nearly half of the cohort had four or more long‐term conditions, while two‐thirds experienced mild/severe frailty. Only 6% were receiving ongoing diabetes care from secondary care diabetes specialist services. For patients with CKD not receiving dialysis, comparing pre‐ and post‐intervention, there were improvements in HbA1c (−13.0 mmol/mol, *p* < 0.001), SBP (−13.7 mm Hg, *p* < 0.0001), and weight (−2.9 kg, *p* < 0.0001). Furthermore, there was an increase in guideline‐directed therapies, with notable usage of SGLT2i (62.9%) and GLP1‐RA (28.4%), while access to diabetes technology increased to 89%.

**Conclusion:**

This new model of care resulted in improved metabolic outcomes, increased utilisation of guideline‐directed therapies, and enhanced access to diabetes technologies. However, the model also revealed significant unmet clinical needs in areas such as access to diabetes care, diabetes eye screening and foot surveillance.

**What's new**

- We highlight a policy implementation gap in diabetes care for individuals with diabetes and advanced CKD, and propose a solution of a new diabetes model of care.
- This new model of care demonstrated notable improvements in metabolic outcomes.
- We believe the new model has signficantly reduced health inequalities by reducing disparties in acess to diabetes care and enhancing access to guideline‐directed theraphies and technologies.

## 1 INTRODUCTION

Diabetes is recognised as a common cause of chronic kidney disease (CKD) and a significant risk factor for the overall mortality, cardiovascular complications, and the onset of end stage kidney disease (ESKD).^1^ In the United Kingdom, diabetes constitutes the primary cause of kidney disease in 30.2% of patients requiring kidney replacement therapy.^2^ Despite its association with poor health outcomes, there exists considerable variation in the provision of diabetes care and a lack of integrated diabetes kidney care for people with advanced CKD.^3^

In the United Kingdom, diabetes care service delivery models are organised into four main tiers, encompassing both primary care and enhanced diabetes care.^4^ These tiers range from the provision of specialist diabetes care for complex cases in the community to advanced specialist care in a secondary care setting. Tier 4, Diabetes Care (Secondary Care Trust), encompasses diverse populations, including inpatient diabetes, foot diabetes MDT, Type 1 diabetes, antenatal diabetes, and children with diabetes, as well as in theory those with CKD stages 4 and 5, and those on renal replacement therapy.^5^ However, while people with complex diabetes care needs without kidney problems are receiving care in secondary care as recommended, there is a mismatch particularly for people with diabetes and advanced CKD (stages 4 & 5) and those undergoing haemodialysis, as most of their kidney care is provided in renal satellite units and with limited or no secondary care diabetes support. The Joint British Diabetes Societies guidelines (JBDS)^6^ highlight these organisational difficulties faced by people with diabetes and advanced CKD in accessing diabetes care. They recommend assigning a named diabetes specialist nurse for each dialysis unit.

In March 2023, we introduced a new diabetes care model for individuals with advanced kidney disease in one of our kidney satellite units in North Central London (NCL). This initiative was facilitated by deploying a senior diabetes specialist nurse with a specialised interest in kidney disease to the renal satellite unit. The purpose of this endeavour was to enhance the capacity of the existing CKD service to provide comprehensive diabetes care for individuals with diabetes and advanced CKD stages 4 and 5, as well as those undergoing haemodialysis. To mitigate the diabetes‐related worsening of vascular complications in this population, the nurse implemented a multifaceted approach that addresses both diabetes and multiple cardiovascular risk factors. The aim of this paper is to describe how this joint diabetes renal model of care meets the diverse needs of individuals with diabetes and advanced CKD.

## 2 METHODS

### 2.1 Study population and the study design

This was a prospective audit conducted at a single centre, encompassing the entire adult population (≥18 years) with diabetes and advanced CKD attending a new expanded diabetes and kidney disease service at a North Central London Renal satellite Unit from March 2023 to February 2024. This audit was approved by the Royal Free London Foundation Trust's Audit Board, with the audit registration number RFH_66422/23.

We used a previously developed algorithm for determining diabetes diagnosis, which was based on either the date a person first received a diagnostic record or if diabetes therapy was prescribed, or if they had an HbA1c reading ≥48 mmol/mol.^5^, ^6^

### 2.2 The audit's objectives were to

- Characterise people with diabetes and advanced CKD who were receiving kidney care at North London renal satellite unit prior to receiving care using the new joint diabetes renal care model.
- To evaluate the extent to which people with diabetes and advanced kidney disease are receiving care that complies with a range of quality metrics specified in the NICE Diabetes^7^ and CKD guidelines,^8^ along with guidelines provided by the Association of British Clinical Diabetologists^9^ and Joint British diabetes society.^3^
- To determine the level of diabetes clinical care required to manage individuals with diabetes and advanced CKD effectively and in a timely manner.

### 2.3 Audit metrics were

- Proportion of patients completed the nine diabetes care processes at the baseline and 12 months. A documentation of blood pressure (BP), cholesterol, creatinine, HbA1c levels, urine albumin–creatinine ratio (uACR), weight, height, foot checks, and diabetic eye screening within the last 12 months.
- Changes in metabolic outcomes (HbA1c, BP, cholesterol levels, weight) from the baseline and 12 months.
- Changes in NICE‐approved therapies and technologies. from the baseline to 12 months.
- The number of diabetes Clinical Session required.

### 2.4 Audit data source

Our data management team extracted data at both the baseline and the 12‐month after the start of the intervention utilising various datasets including Electronic patient record (EPR) Vital data, Health Information Exchange (HIE) data, and Summary Care Records. The retrieved data encompassed a wide range of information, including demographic, clinical, and biochemical details, along with clinical activity data such as the number of diabetes clinical sessions attended. Specifically, we collected data on the age, sex, ethnicity, frailty score, comorbidity load, BP, lipid profile, HbA1c, creatinine, eGFR, uACR, weight, height, the body mass index (BMI), index of multiple deprivation (IMD), and records of current medication prescriptions.

Moreover, we documented information about previous consultations with the specialist diabetes team. Data on current medications were extracted from prescription records over the previous year: for SGLT2i inhibitors and GLP1‐RA, as well as medications related to diabetes, lipid‐lowering, and BP management. Additionally, we gathered data on whether individuals underwent diabetic retinal screening and foot surveillance checks within the preceding 18–24 months. This information was cross‐referenced with GP record coding for diabetic retinopathy and annual check codes to ensure accuracy and completeness.

Ethnicity was categorised into five groups: White, Black, Asian, Other ethnic group and Unknown. The BMI was calculated using the formula BMI = kg/m^2^ and categorised into three groups: ≤25, 25 to <30, and ≥30, broadly indicating healthy weight, overweight, and obesity, respectively. The patient‐level IMD was used as a measure of deprivation, categorised into quintiles (1 being the most deprived and 5 the least deprived).

Aside from diabetes and advanced CKD, we generated a variable entitled ‘comorbidity count’ capturing the count of additional diagnoses (asthma, arterial fibrillation, cancer, chronic obstructive pulmonary disease, dementia, epilepsy, chronic liver disease, hypertension, heart failure, ischaemic heart disease, myocardial infarction, peripheral vascular disease, rheumatoid arthritis, stroke, thyroid disease, and severe mental health disorders (including bipolar disorder and schizophrenia)).

### 2.5 Intervention

In the renal satellite unit in North Central London, a senior diabetes nurse implemented a multifaceted approach encompassing self‐management education (education on clinical targets, setting individualised clinical goals, and providing information on foot care and diabetes eye care), lifestyle advice (exercise, diet, carb awareness, or principles of carb counting), medication optimisation (adjusting, escalating, or de‐escalating treatment), cardiovascular disease prevention (introducing NICE‐approved therapies to reduce cardiovascular burden), and psychosocial support (using motivational interviewing techniques to reduce psychological distress).

The senior nurse ran an independent, nurse‐led low clearance diabetes clinic weekly, conducted a weekly haemodialysis ward round, and supported weekly low clearance multidisciplinary case discussions with a senior consultant nephrologist, renal psychologist, renal dietitian, and CKD nurses. Additionally, she provided joint renal diabetes consultations with senior nephrologists or CKD nurses in the unit. Information on the senior nurse's educational and clinical experience is provided in Appendix 1.

The intervention was delivered over a 12‐month period in hybrid virtual or in‐person sessions, with targeted one‐to‐one support. The key objectives of the intervention were to improve treatment targets (HbA1c, BP, cholesterol), enhance the achievement of diabetes care processes, and increase the uptake of and access to NICE‐approved therapies and technologies. The Senior Nurse assessed and implemented individualised care plans for each patient, equipping them with the knowledge to mitigate the risk of developing acute diabetes complications or worsening vascular complications. She assessed the patient's knowledge related to diabetes care, provided education, and was responsible for teaching certain skills, such as initiating continuous glucose monitoring (CGM) and administering insulin/ other injectable therapies.

Additionally, the nurse was responsible for coordinating care for individuals by liaising with other agencies such as social workers, district nurses, and the wider care team to improve health outcomes.

### 2.6 Statistical analyses

All analyses were conducted using STATA. Categorical variables are presented as percentages, while continuous variables are expressed as mean ± standard deviation. The significance of the difference in means at the baseline and at the 12‐month follow‐up was tested using a paired samples *t*‐test. Data regarding diabetes technology and therapies were also reported as percentages.

## 3 RESULTS

A total of 497 people with advanced CKD received kidney care at St. Pancras renal satellite unit between March 2023 and February 2024.

**FIGURE 1:**
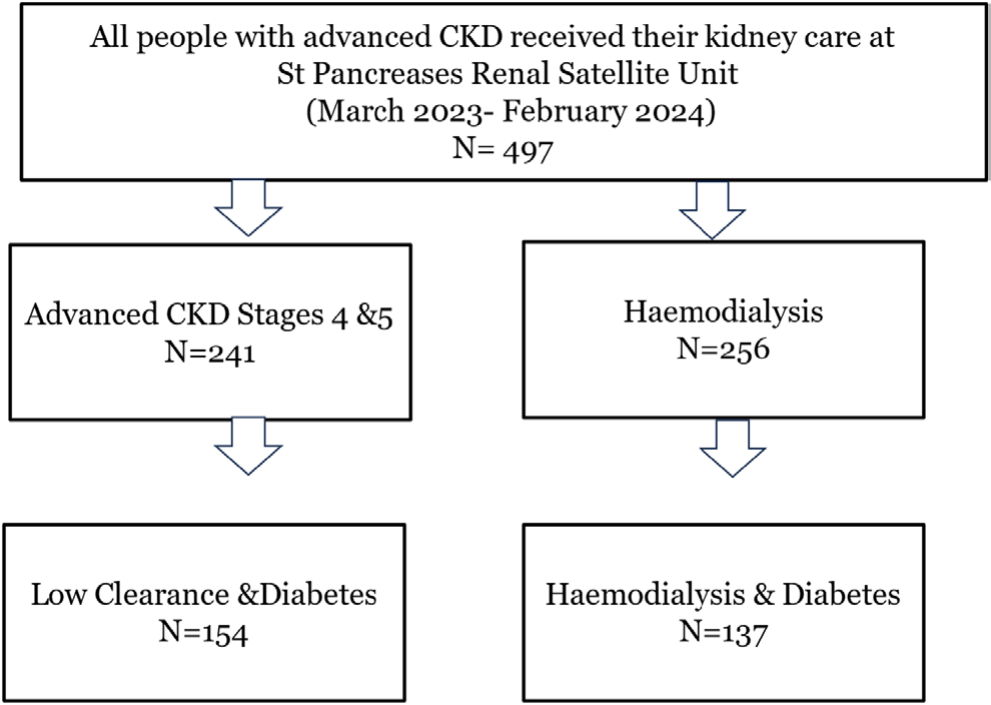
Flow chart.

Among these, 291 (60.2%) had diabetes 277 (95.2%) had type 2 diabetes. During follow up, 1 patient was discharged from the service due to improved kidney function, 3 patients (0.6%) had a kidney transplant, and 16 (3.2%) died before the 12‐month visit. The breakdown of the population is summarised in Figure 1.

Across the entire study population, the mean age of the diabetes advanced CKD cohort was 68.5 (±13.0) years, with 58.4% being male. Approximately half (47.1%) of the cohort lived with four or more other long‐term conditions, and 66.5% of patients had been observed to have mild to severe frailty. Approximately three quarters (73.1%) resided in the most deprived parts of the borough. The ethnic mix reflected the local clinic population with breakdown White (23.7%), Black (18.2%), Asian (12.3%), Other ethnic groups (18.2%) and 27.5% people had unknown. Patients undergoing haemodialysis were younger, had a higher morbidity load, and were frailer and hypertensive compared to the pre‐dialysis patient cohort. Conversely, the pre‐dialysis patient cohort was observed to have suboptimal glycaemic control and high prevalence of obesity (Table 1).

**TABLE 1:** Baseline characteristics of people with diabetes and advanced CKD, who are receiving kidney care at North central satellite unit.

Variables	Categories	Missing	Pre‐dialysis CKD with diabetes (*N* = 154)	Haemodialysis with diabetes (*N* = 137)
Age			72.1 (±11.7)	64.7 (±13.4)
Sex	Male		88 (57.5)	80 (58.5)
	Female		65 (42.5)	57 (41.5)
Ethnicity	White		39 (25.3)	31 (22.6)
	Black		15 (9.7)	37 (27.0)
	Asian		16 (10.4)	20 (14.6)
	Other ethnic		26 (16.9)	27 (19.7)
	Unknown		58 (37.6)	22 (16.1)
BMI	<25	0.9%	21 (13.6)	36 (26.3)
	25–29.9		50 (32.9)	38 (27.7)
	≥30		81 (52.6)	63 (46.0)
IMD	1 (Most deprived)		44 (28.7)	48 (35.6)
	2		70 (45.8)	51 (37.8)
	3		28 (18.9)	20 (14.8)
	4		8 (5.9)	8 (5.9)
	5 (Least deprived)		3 (2.0)	8 (5.9)
Frailty score	<4	9.2%	67 (44.8)	27 (19.7)
	≥4		71 (46.7)	110 (80.3)
Comorbidity count	2–3		32 (20.8)	30 (21.9)
	4–5		68 (44.2)	69 (50.4)
	≥6		54 (35.1)	38 (27.7)
Type of diabetes	Type 1		3 (1.9)	9 (6.6)
	Type 2		151 (98.1)	128 (93.4)
Baseline HbA1c (mmol/mol)			69.4 (±26.5)	55.3 (±23.2)
Baseline CKD stages	Stage 3b		8 (5.2)	–
	Stage 4		103 (66.9)	
	Stage 5		43 (27.9)	
Baseline uACR (mg/mmol)	A1	3.3%	9 (5.8)	–
	A2		51 (33.1)	
	A3		89 (57.8)	
Baseline systolic BP (mmHg)			149.4 (±21.4)	154.9 (±23.1)
Baseline diastolic BP (mmHg)			76.2 (±10.1)	76.1 (±13.6)
Baseline total Cholesterol (mmol/mol)			4.0 (±1.5)	3.8 (±1.2)
Diabetic Retinal screening	Yes		67 (43.5)	55 (40.0)
	No		87 (56.5)	82 (60.0)
Foot check	Yes		71 (46.1)	49 (32.5)
	No		83 (53.9)	88 (58.2)
Mean diabetes clinical sessions provided			2.0 (±1.9)	1.4 (±1.0)
Diabetes provider	GP		146 (94.8)	126 (92.0)
	Secondary care diabetes specialist services		8 (5.2)	11 (8)

### 3.1 Diabetes process of care

All 291 patients with diabetes and advanced kidney disease, regardless of whether they were on dialysis, had their creatinine levels, HbA1c, and BP measured within the previous year. With few exceptions (99%), they also had serum cholesterol levels assessed and BMI recorded (98%). However, within the same timeframe, only 41.9% underwent diabetic eye screening. A significant proportion of those (97 individuals, or 80%) were receiving ophthalmology interventions for severe retinopathy or maculopathy at secondary care, while the remaining 20% were discharged back to regular screening Additionally, only 58.7% had their foot inspections. Among the 154 individuals with diabetes in the pre‐dialysis cohort, 93.4% had albumin‐to‐creatinine ratio (ACR) assessments within the past year.

In the pre‐dialysis cohort, 117 individuals (76%) were prescribed statins, and 113 individuals (73.4%) were prescribed RAAS inhibitors within the previous year. For the haemodialysis population, 111 individuals (81.0%) were prescribed statins, and 107 individuals (78.1%) were prescribed RAAS inhibitors within the previous year.

### 3.2 Diabetes care provision

In the overall cohort of 291 patients, only 6.5% were receiving ongoing diabetes care from secondary care diabetes specialist services.

With implementation of the new service model, the required mean number of diabetes clinical sessions provided by senior specialist diabetes CKD nurses for the pre‐dialysis population was 2.0 (±1.9), and for the haemodialysis population was 1.4 (±1.0). The diabetes clinic appointments ranged from 1 to 12 sessions per patient. All of the haemodialysis population were reviewed in person, whereas pre‐dialysis reviews were conducted through hybrid virtual and in‐person appointments.

At the baseline, 12 (8.1%) patients were on GLP‐1RA and 13 (9.3%) were on SGLT‐2i. By the 12‐month follow‐up, the number of patients receiving GLP‐1RA increased to 54 (36%), while 78 (72.2%) were on SGLT‐2i (Table 2).

**TABLE 2:** Changes in metabolic outcomes and guideline directed diabetes therapies in low clearance population (*N* = 154).

Variables	Baseline	9–12 month	*p*‐Value
HbA1c (mmol/mol)	69.4 (±26.5)	56.4 (±16.5)	*p* < 0.0001
Systolic BP (mmHg)	149.4 (±21.4)	135.7 (±18.5)	*p* < 0.0001
Diastolic BP (mmHg)	76.2 (±10.1)	72.6 (±10.8)	*p* = 0.0002
Total cholesterol (mmol/L)	4 (±1.15)	4.0 (±1.3)	*p* = 0.81
Weight (kg)	85.1 (±20.3)	82.2 (±19.8)	*p* < 0.0001
SGLT2i (Eligible *n* = 108)	13 (9.3%)	78 (72.2%)	
GLP1‐RA (Eligible *n* = 148)	12 (8.1%)	54 (36.5%)	

Access to diabetes technology for glycaemic management was limited, with only 4.5% of those eligible having a flash or continuous glucose monitor (CGM) at the baseline, which increased to 89% at the 12‐month follow‐up for individuals with diabetes undergoing haemodialysis and treated with insulin therapy (see Table 3).

**TABLE 3:** Metabolic outcomes and NICE approved technology in Haemodialysis population (n = 137).

Variables	Baseline	12 months	*p*‐Value
HbA1c	55.3 (±23.2)	49.6 (±15.2)	*p* = 0.004
Systolic BP	154.9 (±23.1)	137.0 (±23.3)	*p* < 0.0001
Diastolic BP	76.1 (±13.6)	65.8 (±13.1)	*p* < 0.0001
Total cholesterol	3.8 (±1.2)	3.6 (±1.00)	*p* = 0.0001
Access to diabetes technology individuals treated on insulin (*N* = 66)	3 (4.5%)	59 (89.3%)	

### 3.3 Metabolic outcomes for peri dialysis population

HbA1c levels decreased by 13 mmol/mol over the 12‐month follow‐up period. During this time, patients experienced significant reductions in body weight (2.9 kg), as well as in systolic (13.9 mmHg) and diastolic BP (3.5 mmHg). Despite showing a trend of improvement in cholesterol levels, the whole cohort did not exhibit significant changes compared to the baseline (Table 2).

### 3.4 Metabolic outcomes in haemodialysis population

Mean glycated haemoglobin (HbA1c) was 55.3 (±23.2) with clear evidence for a marked (*p* < 0.004) reduction in the HbA1c at the 12‐month follow‐up (49.6 ± 15.1). There were significant reductions in BP (systolic drop of approximately 18 mmHg), and serum total cholesterol (0.2 mmol/L, *p* = 0.0001) in the dialysis population (Table 3).

## 4 DISCUSSION

We have shown clear unmet care need in people with diabetes and advanced kidney disease. Addressing this need was associated with significant improvements in metabolic outcomes, increased access to NICE‐approved therapies, and diabetes technologies over a 12‐month period. The adoption of novel care models such as ours is important for more effective implementation of guidelines into practice.

The socio‐clinical demographic data reveals the complex health needs of the cohort, marked by a high prevalence of multimorbidity, obesity, frailty, and deprivation. This underscores the necessity for a more integrated, coordinated, and multispecialty care model to effectively address their health needs.

We have demonstrated that individuals with diabetes and advanced CKD treated at a Renal Satellite Unit have a higher attainment of recorded diabetes care processes at the baseline. Many of these results surpass those reported by the National Diabetes Audit (NDA) for people with type 2 diabetes in England and Wales.^10^ This suggests that nephrologists looking after this cohort are well‐equipped to investigate and stratify cardiovascular risks. However, the data also revealed significant unmet clinical needs regarding foot surveillance and diabetic retinopathy screening. A similar result was reported by Cushley and colleagues, indicating that 15% of people with diabetes undergoing haemodialysis never attended the national diabetes eye screening program. To address this issue, they introduced handheld retinal imaging at the haemodialysis unit. Subsequent screening revealed that 23% had sight‐threatening diabetic retinopathy (R2, R3A, R3S) in their worst eye, and 11% had maculopathy.^11^

Individuals with diabetes and advanced CKD represent a high‐risk group more likely to present with sight‐threatening retinopathy.^12^ However, the NCL ICS has some of the lowest retinopathy screening rates, resulting in consultant referrals. Provision of eye care would need to be relooked at to prevent poor outcomes in this cohort. The figures on foot surveillance are concerning, especially considering that people on haemodialysis have a five times higher risk of foot ulceration compared to the pre‐dialysis population.^13^ The JBDS^3^ guideline suggests at least monthly foot checks for people with diabetes on haemodialysis.

Our findings demonstrate that the existing system for arranging specialist diabetes care is suboptimal for people with diabetes and advanced CKD. Joseph et al.^14^ also found that, in a population of 225 people with diabetes on haemodialysis across three North West London dialysis units, only 80 were under specialist diabetes care. This finding was echoed by Wijewickrama et al.,^15^ who reported variation in access to diabetes specialist care for people undergoing peritoneal dialysis. This suggests a need to reexamine the national diabetes care model for this population, despite their advanced renal disease and complicated diabetes. These individuals are falling through the cracks as they often have a restricted lifestyle due to the demands of dialysis sessions, leaving little time for other appointments.

This new model of care was associated with greater adoption of guideline‐directed therapies and diabetes technologies. Our data demonstrated the underutilisation of newer NICE‐approved therapies like GLP1RA and SGLT2‐i at the baseline, with a significant increase at the 12‐month point. This increase will have implications for improving outcomes and increasing eligibility for kidney transplantation in this cohort. Additionally, our data revealed disparities in access to diabetes technologies among people with diabetes undergoing haemodialysis and treated with insulin. According to the NHS England guidance^16^ on the implementation of Libre glucose monitoring prescribing across the NHS in London, patients with any form of diabetes undergoing haemodialysis and receiving insulin treatment are eligible for Libre glucose monitoring. However, despite this guidance, only 4.5% of eligible patients had been established on the Libre glucose monitoring system at the baseline. Additionally, 56 more patients were established on the Libre system at the 12‐month follow‐up. These technologies offer real‐time insights into glucose pattern recognition, aiding in management and, in some ways, reducing the burden of diabetes. In a retrospective analysis utilising US administrative claims data, Hannah et al.,^17^ found that the implementation of CGM significantly reduced rates of hospitalisations related to diabetes‐induced hypoglycaemia or hyperglycaemia in insulin‐treated individuals with type 2 diabetes and CKD.

Several observational studies and audits utilising both pre‐ and post‐designs have investigated joint diabetes and kidney clinics. These interventions have been shown to slow the decline in kidney function and enhance the achievement of clinical goals, such as HbA1c levels.^18^, ^19^, ^20^, ^21^, ^22^, ^23^ Our integrated joint diabetes and renal clinic was among the first to provide such a service in a community renal satellite unit serving the population with diabetes and advanced CKD (Stages 4 & 5), including those on haemodialysis.

The NHS long‐term plan^24^ prioritises identifying and addressing missed elements in care pathways, alongside improving out‐of‐hospital care through multidisciplinary teams and digital healthcare services. Our care model, led by senior diabetes specialist nurses with advanced skills in managing diabetes and CKD, fulfils these requirements. It combines face‐to‐face and virtual reviews, guided by data‐driven care delivery principles.

### 4.1 Limitations

Our study has several limitations. First, the study's single‐centre design limits its external validity and broader clinical application. However, we anticipate that similar results would be obtained in other renal satellite units. Another limitation includes the accuracy of HbA1c in assessing glycaemic status in people with diabetes and advanced CKD. On the other hand, our study provides new insights into the gaps in policy and clinical care, calling for policy changes to provide the diabetes care that people with advanced CKD require. We are in the process of conducting a matched analysis to compare outcomes between patients receiving the integrated care model and those receiving standard care by creating matched cohorts.

## 5 CONCLUSIONS

Our data demonstrate that patients who attended the new integrated diabetes and kidney model of care for people with diabetes and advanced CKD benefited from the availability of diabetes expertise. This model of care was associated with improvements in metabolic outcomes, increased use of guideline‐directed therapies, and enhanced access to diabetes technologies. Our findings also indicate that specialist diabetes nurses can effectively work in renal satellite units to address the complex diabetes needs of individuals with advanced CKD. Additionally, the model has revealed a significant burden of unrecognised and unmet clinical needs in this cohort. Implementing this model of care could help bridge the gap in policy and clinical care for people with diabetes and advanced CKD.

## FUNDING INFORMATION

AstraZeneca (AZ) has provided support for the project under joint working agreement with Royal Free London. AZ was not involved in the study design; the collection, analysis, and interpretation of data; the writing of the report, or the decision to submit the article for publication. The manuscript reflects only the author___s view.

## CONFLICT OF INTEREST STATEMENT

The authors HH‐A received speaker honoraria from AstraZeneca and Bayer. MR received speaker honoraria from AstraZeneca. DN on the board of UCL Partners, and the UK Kidney Association Director of Informatics Research. DW has an ongoing consultancy contract with AstraZeneca. He has received payments for consultancy working and/or speaking activities from Amgen, Astellas, Bayer, Boehringer Ingelheim, Eledon, GSK, Galderma, Gilead, Janssen, Mundipharma, Menarini, MSD, NovoNordisk, Pharmacosmos, Tricida and Vifor

## Data Availability

Data are available from the first author upon reasonable request.

## APPENDIX 1

Senior Diabetes Nurse Profile

**Qualifications:**

- PhD in Epidemiology
- MSc in Diabetes Care
- BSc in Adult Nursing
- Non‐medical prescribing
- Leadership
- System leader

**Grade AFC: 8C**

- Lead Clinical Academic Research Nurse in Diabetes & CKD

**Experience:**

- Extensive experience delivering specialised diabetes nursing care in both outpatient and inpatient settings.
- Substantial experience in managing and leading specialty nursing services.
- Over the years, she has led and established a nurse‐led renal diabetes clinic, managing various specialised areas such as low clearance diabetes clinic, kidney transplant diabetes clinic, haemodialysis nurse‐led ward round, and inpatient management of diabetes in the renal ward.

**Research and Academic Contributions:**

- Involved in a number of studies in the field of: Diabetes Kidney diseases & Diabetic retinopathy.
- Core member of the Joint British Diabetes Societies (JBDS) Haemodialysis Working Group.
